# Supplementary material for: A Multi-Parametric Approach for Characterising Cerebral Haemodynamics in Acute Ischaemic and Haemorrhagic Stroke
Source: Healthcare (Basel). 2024 May 8;12(10):966. doi: 10.3390/healthcare12100966 (PMC11120760; doi:10.3390/healthcare12100966)
Supplement: Supplementary file 1 [file healthcare-12-00966-s001.zip › Table S1.pdf]

Table S1: Spectral power density of ABP and CBv

Parameters	AIS<br>n = 68	ICH<br>n = 12	P-value
CBv power LF range (AH), cm/s^2	0.18 ± 0.34	0.25 ± 0.22	0.50
CBv power MF range (AH), cm/s^2	0.02 ± 0.04	0.05 ± 0.08	0.20
CBv power HF range (AH), cm/s^2	0.01 ± 0.03	0.02 ± 0.04	0.34
ABP power LF range (AH), $mmHg^2$	0.54 ± 0.54	0.38 ± 0.39	0.34
ABP power MF range (AH), $mmHg^2$	0.09 ± 0.23	0.10 ± 0.21	0.92
ABP power HF range (AH), $mmHg^2$	0.02 ± 0.03	0.02 ± 0.03	0.93
CBv power LF range (UH), cm/s^2	0.28 ± 0.33	0.25 ± 0.22	0.77
CBv power MF range (UH), cm/s^2	0.03 ± 0.04	0.05 ± 0.06	0.29
CBv power HF range (UH), cm/s^2	0.01 ± 0.02	0.019 ± 0.015	0.68
ABP power LF range (UH), $mmHg^2$	0.54 ± 0.53	0.43 ± 0.38	0.48
ABP power MF range (UH), $mmHg^2$	0.07 ± 0.10	0.06 ± 0.09	0.80
ABP power HF range (UH), $mmHg^2$	0.02 ± 0.02	0.01 ± 0.01	0.79

CBv, cerebral blood flow velocity; ABP, arterial blood pressure; AH, affected hemispheres; UH, unaffected hemispheres. LF, low frequency; MF, medium frequency; HF, High frequency.

P-values for difference between ischemic and haemorrhagic stroke.
