# Supplementary material for: A Multi-Parametric Approach for Characterising Cerebral Haemodynamics in Acute Ischaemic and Haemorrhagic Stroke
Source: Healthcare (Basel). 2024 May 8;12(10):966. doi: 10.3390/healthcare12100966 (PMC11120760; doi:10.3390/healthcare12100966)
Supplement: Supplementary file 1 [file healthcare-12-00966-s001.zip › Table S2.pdf]

Table S2: Cerebral autoregulation and baroreceptor sensitivity parameters

Parameters:	AIS<br>n = 68	ICH<br>n = 12	P- value
CBv (AH), cm/s	39.74 ± 15.46	49.54 ± 19.28	**0.05**
CBv (UH), cm/s	43.91 ± 13.67	46.49 ± 13.22	0.54
SYSTOLIC VEL, (UH), cm/s	69.64 ± 18.84	77.38 ± 16.77	0.18
DIASTOLIC VEL, (UH), cm/s	27.32 ± 9.97	30.42 ± 7.68	0.31
Mean coherence (AH)	0.59 ± 0.25	0.47 ± 0.20	0.12
ARI (AH)	5.11 ± 1.87	5.77 ± 2.08	0.28
NMSE, (AH)	0.18 ± 0.14	0.20 ± 0.11	0.64
Step response recovery (%) at 4 s, (AH)	64.44 ± 31.13	82.57 ± 35.55	0.07
Mean LOWER conf. limit coherence, (AH)	0.47 ± 0.30	0.31 ± 0.21	0.10
Mean UPPER conf. limit coherence, (AH)	0.70 ± 0.21	0.63 ± 0.21	0.30
LF gain (AH), % / mmHg	0.33 ± 0.18	0.37 ± 0.18	0.52
MF gain (AH), % / mmHg	0.54 ± 0.35	0.53 ± 0.24	0.93
HF gain (AH), % / mmHg	0.65 ± 0.33	0.78 ± 0.45	0.22
LF phase (AH), radians	0.59 ± 0.64	0.86 ± 0.83	0.20

MF phase (AH), radians	0.57 ± 0.40	0.46 ± 0.41	0.38
HF phase (AH), radians	0.06 ± 0.27	0.16 ± 0.34	0.26
Mean coherence (UH)	0.57 ± 0.27	0.41 ± 0.28	0.07
NMSE (UH)	0.17 ± 0.12	0.28 ± 0.16	0.24
Mean Coherence (VLF), (UH)	0.43 ± 0.21	0.32 ± 0.20	0.10
Mean Coherence (LF), (UH)	0.50 ± 0.23	0.28 ± 0.18	0.002
Mean Coherence (HF), (UH)	0.55 ± 0.25	0.37 ± 0.23	0.02
Step response recovery (%) at 4 s, (UH)	68.16 ± 24.34	79.09 ± 22.45	0.15
Mean LOWER conf. limit coherence, (UH)	0.45 ± 0.31	0.29 ± 0.28	0.08
Mean UPPER conf. limit coherence, (UH)	0.69 ± 0.23	0.55 ± 0.28	0.06
LF gain (UH), % / mmHg	0.51 ± 0.44	0.36 ± 0.28	0.25
MF gain (UH), % / mmHg	0.64 ± 0.47	0.43 ± 0.26	0.14
HF gain (UH), % / mmHg	0.75 ± 0.43	0.71 ± 0.44	0.73
LF phase (UH), radians	0.62 ± 0.52	1.12 ± 0.60	**0.004**
MF phase (UH), radians	0.60 ± 0.34	0.54 ± 0.27	0.53
HF phase (UH), radians	0.05 ± 0.26	0.20 ± 0.32	0.09

Mean gain HF range (BRS)	9.04 ± 9.37	5.94 ± 7.75	0.28
total power (PI), ms^2	2011.7 ± 3918.01	1201.8 ± 2462.1	0.44
total power (SBP), $mmHg^2$	33.08 ± 27.43	32.04 ± 26.93	0.77
VLF power (PI), ms^2	313.58 ± 406.4	138.71 ± 115.2	0.13
VLF power (SBP), $mmHg^2$	15.65 ± 15.29	14.18 ± 14.01	0.75
BRS for VLF band	4.56 ± 2.77	2.98 ± 2.22	0.06
LF power (PI), ms^2	523.68 ± 1155.43	283.67 ± 546.03	0.44
LF power (SBP), $mmHg^2$	7.67 ± 6.84	5.31 ± 3.36	0.18
HF power (PI), ms^2	1475.91 ± 3216.4	1539.5 ± 3751.1	0.52
HF power (SBP), $mmHg^2$	4.96 ± 10.79	7.9 ± 12.6	0.30
BRS for HF band	10.61 ± 7.56	9.06 ± 10.03	0.53
alpha index	8.82 ± 5.72	6.58 ± 5.95	0.22

AH, affected hemispheres; UH, unaffected hemispheres; VLF, very low frequency LF, low frequency; MF, medium frequency; HF, High frequency; CBv, Cerebral Blood Velocity; VEL, Velocity; ARI, Autoregulation index; NMSE, normalized mean-square error; BRS, baroreceptor sensitivity; PI, Pulse Interval; SBP; systolic blood pressure, alpha index (Average BRS LF + BRS HF).

P-values for difference between ischemic and haemorrhagic stroke.
