# Supplementary material for: A Multi-Parametric Approach for Characterising Cerebral Haemodynamics in Acute Ischaemic and Haemorrhagic Stroke
Source: Healthcare (Basel). 2024 May 8;12(10):966. doi: 10.3390/healthcare12100966 (PMC11120760; doi:10.3390/healthcare12100966)
Supplement: Supplementary file 1 [file healthcare-12-00966-s001.zip › Table S3.pdf]

Table S3: Significant cerebral haemodynamic and baroreceptor sensitivity parameters

Parameters:	AIS<br>n = 68	ICH<br>n = 12	P- value	95% CI	Difference between means ± SEM
SYSTOLIC VEL, (AH), cm/s	64.39 ± 17.36	82.87 ± 27.90	0.01	(7.516; 33.74)	20.63 ± 6.570
DIASTOLIC VEL, (AH), cm/s	26.03 ± 8.66	32.51 ± 9.86	0.05	(- 0.1009; 14.50)	7.198 ± 3.659
Mean Coherence (VLF), (AH)	0.43 ± 0.18	0.28 ± 0.16	0.01	(- 0.2934; - 0.03816)	-0.1658 ± 0.06398
Mean Coherence (LF), (AH)	0.50 ± 0.21	0.34 ± 0.17	0.01	(- 0.3246; - 0.04244)	-0.1835 ± 0.07073
Mean Coherence (HF), (AH)	0.56 ± 0.21	0.39 ± 0.18	0.016	(- 0.3113; - 0.03247)	-0.1719 ± 0.06988
ARI (UH)	4.81 ± 2.02	5.96 ± 1.77	0.07	(- 0.09844; 2.197)	1.050 ± 0.5764
Mean Coherence (LF), (UH)	0.51 ± 0.23	0.28 ± 0.18	0.002	(- 0.3706; - 0.08491)	-0.2278 ± 0.07173
Mean Coherence (HF), (UH)	0.55 ± 0.22	0.37 ± 0.23	0.02	(- 0.3363; - 0.02705)	-0.1817 ± 0.07762
Mean gain MF range (BRS)	5.89 ± 5.32	2.71 ± 1.89	0.046	(- 6.323; - 0.05268)	-3.188 ± 1.575
BRS for LF band	6.7 ± 4.2	4.10 ± 2.13	0.040	(- 5.091; - 0.1118)	-2.601 ± 1.250
